# High-quality metagenomic DNA from marine sediment samples for genomic studies through a preprocessing approach

**DOI:** 10.1007/s13205-016-0482-y

**Published:** 2016-08-06

**Authors:** Solly Solomon, Bhavya Kachiprath, G. Jayanath, T. P. Sajeevan, I. S. Bright Singh, Rosamma Philip

**Affiliations:** 1 Department of Marine Biology, Microbiology and Biochemistry, School of Marine Sciences, Cochin University of Science and Technology, Kochi, 16 Kerala India; 2 National Centre for Aquatic Animal Health, Cochin University of Science and Technology, Fine Arts Avenue, Kochi, 16 Kerala India

**Keywords:** Preprocessing, High molecular weight DNA, DNA isolation, Metagenomic DNA

## Abstract

Recent advances in culture-independent studies of microbes had proved to be more reliable and efficient than the conventional ones. The isolation of good quality and quantity of total community DNA are one of the major hurdles in this endeavour. Shearing of DNA during the extraction process and the co-extraction of inhibitory compounds reduce the quality of the isolated nucleic acids making it unsuitable for the construction of large insert metagenomic libraries. In the present study, a multi-level filtration step was brought in which efficiently isolated total bacterial DNA from three different environment samples. The preprocessing method could efficiently improve the 260/230 ratio of the isolated DNA by 2.3–45 % and decreased the protein contamination by 22.5–34.5 % on saltpan and arctic sediment samples, respectively. The more significant part of the experiment was that the DNA obtained was of high quality with minimal shearing making it most suitable for the construction of large insert genomic libraries. PCR amplification of 16S rRNA gene confirmed that the filtration method was effective in the isolation of high-quality DNA.

## Introduction

Metagenomic approach provides access to microbial genomics and their function which helps in the exploitation of novel biocatalysts from unculturable microbial communities of various ecosystems. Community DNA isolation from different marine environments is a challenging process, since the ecosystems are extremely diverse and contain inhibitory compounds, such as humic acids. Ecosystems, such as mangroves, salt pans, and Arctic deserve, much attention in terms of metagenomic studies due to its uniqueness. Mangroves are productive marine ecosystems and biologically important intertidal zones rich in nutrient content and micro flora. Microbial community found in mangrove sediments is a potent source of important biocatalysts that enable them to live in adverse conditions. Salt pans are manmade ecosystems with extreme conditions, where microorganisms survive at very high salinities with intense solar radiations. Arctic regions are characterized by extremely low temperature, and serve as an important source of isolation of psychrophilic/psychrotolerant microbes, and also cold-adapted enzymes. Metagenomics is a very potent tool for the analysis of these extreme habitats harboring organisms that are often difficult to cultivate.

## Materials and methods

### Sample collection

Surface sediment samples were collected from saltpans (Lat: 15°29′57.12″N and Long: 73°50′49.06″E) of Ribander, Goa and mangrove areas (Lat: 9°54′N and Long: 76°17′60″E) in Cochin, Kerala. Arctic sediment samples (Lat: 78°99′31″N and Long: 12°3′00″E) were collected during the Summer Arctic Expedition of NCAOR (2014) from Kongsfjorden, Arctic. Samples were stored at −80 °C for further analysis.

### Preprocessing of sediment samples

Sediment samples from mangrove, saltpan, and arctic environments were preprocessed to remove humic acid and other inhibitory materials from the samples. Preprocessing involved washing of the sediment samples with filtered sea water by low-speed centrifugation and recovery of the bacteria through filtration. For this, 100 g of the sediment samples were resuspended in 2 l of suspension solution (0.22 μ filtered sea water supplemented with tween 20 at a concentration of 1 ml l^−1^ v/v), mixed well using a magnetic stirrer for 15 min, and centrifuged at 450×*g* for 5 min. A multistep filtration was performed using Whatman™ No. 1 filter paper of pore size 11 μ, followed by 1.2 μ Whatman™ GF/C filter and finally 0.22 μ cellulose nitrate filter (Himedia) membrane. The 0.22 μ filter paper with the residue (the bacterial fraction) was then washed with an extraction buffer [100 mM Tris–HCl, 100 mM EDTA, (pH 8.0), 100 mM sodium phosphate (pH 8.0) and 1.5 M NaCl] supplemented with 0.1 % tween 20 to collect the bacterial biomass. DNA was extracted from the bacterial biomass as per Zhou et al. (1996).

### Environmental DNA extraction

The metagenomic DNA was extracted directly from both preprocessed and non-preprocessed sediment samples by employing Zhou et al. (1996) protocol with modifications. The DNA extraction procedure involved suspending 200 mg sediment sample (wet weight) in 500 μl extraction buffer [100 mM Tris–HCl, 100 mM EDTA, (pH 8.0), 100 mM sodium phosphate (pH 8.0) and 1.5 M NaCl]. To the suspension 50 μl of 10 % CTAB, 50 μl of 20 % SDS and 10 μl Proteinase K (20 ng/μl) was added. The suspension was incubated at 55 °C for 2 h. Subsequently, samples were centrifuged for 10 min at 10,000 rpm, and the supernatants were removed to a new micro centrifuge tubes. The resulting supernatants were pooled and mixed with an equal volume of chloroform: iso-amyl alcohol (24:1, v/v). The aqueous phase was transferred to a new tube after centrifugation at 10,000 rpm for 20 min. To the aqueous phase, 600 μl iso-propanol was added and the mixture was left at 4 °C overnight, followed by high-speed centrifugation (14,000 rpm, 30 min). The DNA pellet was washed with ice-cold 70 % (v/v) ethanol, absolute ethanol, and resuspended in sterile double-distilled water.

### Quality of the DNA

Quality and yield of the isolated DNA were determined. DNA quality was analysed by measuring 260/280 ratio (DNA/protein) and 260/230 ratio (DNA/humic acid) using Hitachi U-2900 spectrophotometer to check contamination by protein and humic acid substances, respectively.

### Gel electrophoresis

DNA samples (3 μl each) were loaded on 0.8 % agarose gel supplemented with ethidium bromide, and electrophoresis was performed at 70 V for 45 min. The gels were visualised using Gel documentation system (BioRad, USA).

### Determination of purity of DNA by PCR

The region encoding 16S rRNA gene (1465 bp) was amplified using universal eubacterial primers 27f (AGAGTTTGATCTGGCTCAG) and 1492r (TACGGYTACCTTGTTACGACTT) to determine whether PCR inhibitors were present in the isolated DNA. PCR was carried out with an initial denaturation at 95 °C for 5 min followed by 35 cycles of denaturation at 94 °C for 45 s, annealing at 58 °C for 45 s, and extension at 72 °C for 1 min with a final extension for 10 min at 72 °C. Visual comparison was done under UV light after electrophoresis of 3 μl each of the amplicons on 1 % agarose using Gel documentation system (BioRad, USA).

## Results

**Fig. 1:**
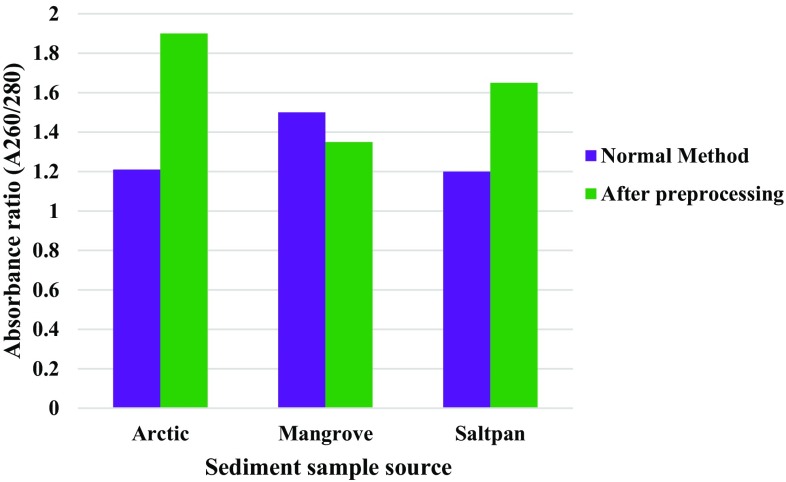
Purity of DNA (A_260_/A_280_) from different marine environments

**Fig. 2:**
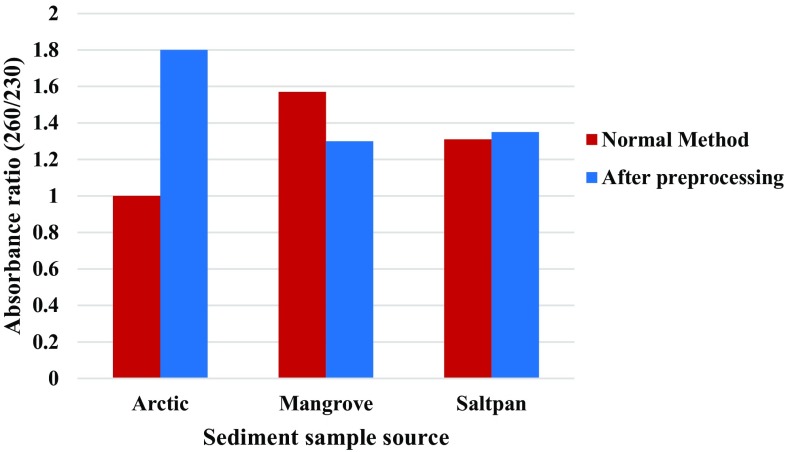
Purity of DNA (A_260_/A_230_) from different marine environments

In the present study, washing of the sediment, followed by a sequential multistage filtration, was carried for the samples prior to the extraction of total genomic DNA following Zhou et al. (1996). DNA isolated after preprocessing of the sediment was compared with that isolated directly without any treatment. Comparative analysis revealed considerable variations in yield and purity of DNA obtained from the different samples (preprocessed and normal). With respect to purity, 260/280 ratio of DNA samples from processed samples were 1.9 and 1.65 for arctic and saltpan samples, respectively, compared to 1.21 and 1.2 obtained by the direct method (Fig. 1). As shown in Fig. 2, DNA from arctic sediment has A_260_/A_230_ ratio close to optimum, indicating DNA with comparatively reduced humic acid content obtained by filtration method.

**Fig. 3:**
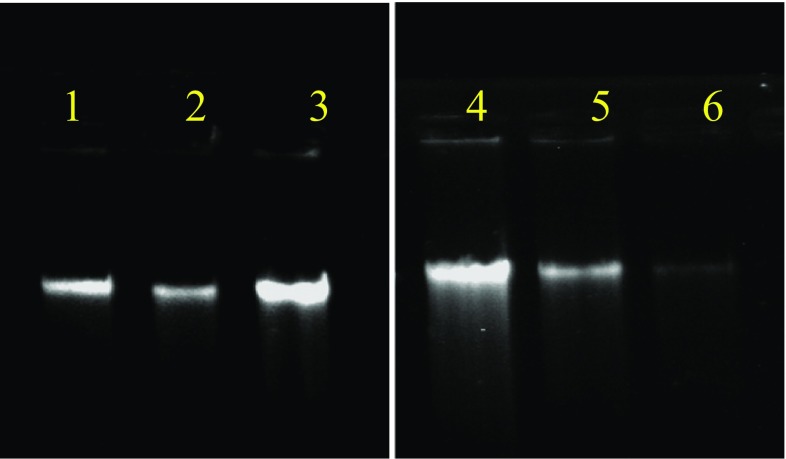
Electrophoretogram of the DNA isolated from various sediment samples (*lanes* *1*–*3*: normal method and *lanes* *4*–*6*: preprocessed): arctic (*lane* *1*), saltpan (*lane* *2*), and mangrove (*lane* *3*); arctic (*lane* *6*), saltpan (*lane* *5*), and mangrove (*lane* *4*)

**Fig. 4:**
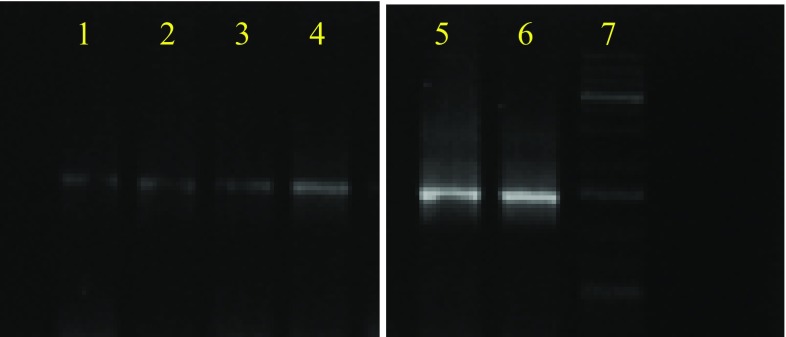
Electrophoretogram of the 16S rRNA gene amplification of isolated genomic DNA (*lanes* *1*–*3*: normal method and *lanes* *4*–*6*: preprocessed): arctic (*lane* *1*), saltpan (*lane* *2*), and mangrove (*lane* *3*); arctic (*lane* *6*), saltpan (*lane* *5*), mangrove (*lane* *4*), and 1 kb ladder (*lane* *7*)

Concentration of the DNA based on the spectrophotometric observations showed that DNA extracted from the preprocessed mangrove sediment yielded 0.779 μg/g sediment while those of saltpan and arctic were 0.476 and 0.088 μg/g, respectively. The yield of DNA from samples without preprocessing was 283.2, 33.25, and 9.69 μg/g for mangrove, saltpan, and arctic sediments, respectively (Table 1). The increase in yield of DNA in the non-processed samples may be due to the presence of eukaryotic organisms with more DNA content, as we had selectively taken only the bacterial fraction in the preprocessed samples. Comparative analysis revealed that the yield of DNA obtained from the preprocessed samples of arctic and saltpan environments was less but with high quality and less humic acid content than DNA isolated directly from the samples (Fig. 3). For mangrove samples, DNA yield was high in the case of direct isolation but with high humic acid content and low quality with high shearing. PCR amplification of 16S rRNA gene was carried out to verify the purity of the isolates, which clearly depicted that the preprocessing of the sediment gave DNA of higher quality as evidenced from the bands on the electrophoretogram (Fig. 4).

**Table 1:** Yield of DNA (μg/g) from different marine environments

Yield of DNA (μg/g)	Arctic	Saltpan	Mangrove
Sediment sample (normal)	9.69	33.25	283.2
Sediment sample (preprocessed)	0.088	0.476	0.779

## Discussion

The sediment samples under the current experiment were rich in microorganisms as well as organic matter especially humic acids which denature DNA by binding phenolic groups to amides. The presence of humic substances in DNA samples, interfere in enzyme-mediated molecular processes, such as digestion, and amplification and ligation in metagenomic library construction (Paul and Clark 1989; Robe 2003; Whitehouse and Hottel 2007). Since downstream processes in molecular biology demand good quantity of inhibitor-free metagenomic DNA, extraction methods have high significance (Siddhapura et al. 2010). Numerous DNA extraction methods in vogue had been discussed and practiced for the isolation of DNA from soil (Bruce et al. 1992; Zhou et al. 1996; Kuske et al. 1998; Yeates et al. 1998; Miller et al. 1999; Bertrand et al. 2005; Desai and Madamwar 2007). However, the major problem is the humic acid contamination and shearing of DNA that makes it unsuitable for the construction of large insert libraries. Use of polyvinyl pyrolidone (PVP), polyvinyl polypyrolidone (PVPP), CTAB, and PEG has been helpful in reducing the load of humic and fulvic acids along with that of DNA, but it significantly reduces the yield. Purdy et al. (1996) used PEG to precipitate DNA which significantly reduced humate contamination, but its yield was significantly less compared to that of alcohol precipitation (Krsek and wellington 1999). Krsek and wellington (1999) also suggested the comparative reduction in the co-extraction of humic substances by the use of low amounts of SDS and EDTA during DNA isolation. Harry et al. (1999) suggested the use of electrophoretic methods to be the best for the separation of humic acid from DNA. The presence of high levels of phenolic compounds in crude DNA extracts even after consistent purification by electrophoresis decreased the PCR amplification. Abu Al-Soud (2000) explained the use of BSA along with the PCR mix to improve the amplification. The use of several columns and beads of Sepharose significantly reduced the humate load, but it significantly reduced the amount of DNA which can lead to a misrepresentation of the microflora in the system. The use of beads and columns also results in extensive physical strain that shears the DNA making it not suitable for the construction of large insert libraries. The use of gel-plus-minicolumn and gel-plus-concentrator methods (Zhou et al. 1996), Sephadex G-200 spin column purification (Miller et al. 1999), and cesium chloride (CsCl) density gradient centrifugation (Bertrand et al. 2005) is laborious, time-consuming, and result in significant DNA loss. Miller et al. (1999) also stated that the serial dilution of DNA reduced the concentration of PCR inhibitors.

The successful recovery of high molecular weight (HMW) DNA and its quality is a mandate for the molecular analysis to access the large pool of genomic information encoded within the metagenome (Ward et al. 1990; Zhou et al. 1996). Construction of large insert metagenomic library is currently used as a genomic approach to study the physiology of unculturable microorganisms (Rondon et al. 2000; Liles et al. 2003). Isolation of HMW DNA is important in the construction of metagenomic libraries, as it increases the possibilities of retaining a complete genetic machinery needed for a biosynthetic pathway (Bertrand et al. 2005). It also helps to reduce the risk of chimera formation during PCR amplification (Liesack and Stackebrandt 1992).

In this study, sediment samples from saltpan, mangrove, and arctic environments were preprocessed to remove inhibitory substances, such as humic acid and fulvic acid, from the sediment samples. The preprocessing steps involved sequential multistage filtration. Sediment samples were treated with saline water, subjected to low-speed centrifugation and filtered through filter membranes to retain the microbes. DNA was extracted from the bacterial fraction on the filter membranes. DNA extracted from this modified filtration method was compared with the DNA samples isolated without any preprocessing. The preprocessing could efficiently improve the 260/230 ratio of the isolated DNA by 2.3–45 % which depicts the reduction in the co-isolation of humic acid and it showed a pronounced decrease in the protein contamination by 22.5–34.5 % on saltpan and arctic sediment samples, respectively. The most significant observation was that preprocessing also helped to reduce the shearing of genomic DNA which is needed for the downstream molecular analyses, such as PCR and large insert genomic library construction without further purification or selection steps. The quality and purity of the metagenomic DNA were evaluated based on the PCR efficacy analysis, as *Taq* polymerase is sensitive to contaminants, such as humic acid (Zhou et al. 1996), and it clearly showed that the preprocessing could yield DNA with a better quality as evident from the strength of the amplicons in comparison with those isolated directly. Earlier reports showed the need of 1000–10,000-fold dilution of metagenomic DNA for a successful amplification of the 16S rRNA gene. Hence, the PCR efficacy analysis also clearly showed that the metagenomic DNA isolated contained relatively low concentration of PCR inhibitory substances and has sufficient purity for PCR without the need for further purification as compared to other DNA extraction methods (Borneman et al. 1996; Zhou et al. 1996; Miller et al. 1999; Bertrand et al. 2005).

## Conclusion

Salient findings include high-quality DNA from arctic and saltpan regions by the preprocessing of the sediment samples. In the present study, the metagenomic DNA isolated (without preprocessing) from sediments were found to have high yield, but high humic acid contamination. Saline washing, centrifugation, and repeated filtration of the sediment samples prior to cell lysis and DNA extraction resulted in the isolation of high molecular weight DNA with moderate yield, high purity with less shearing, and was found suitable for large insert metagenomic library construction.
